# Reconstructing Bronze Age diets and farming strategies at the early Bronze Age sites of La Bastida and Gatas (southeast Iberia) using stable isotope analysis

**DOI:** 10.1371/journal.pone.0229398

**Published:** 2020-03-11

**Authors:** Corina Knipper, Cristina Rihuete-Herrada, Jordi Voltas, Petra Held, Vicente Lull, Rafael Micó, Roberto Risch, Kurt W. Alt

**Affiliations:** 1 Curt Engelhorn Center Archaeometry gGmbH, Mannheim, Germany; 2 Departament de Prehistòria, Universitat Autònoma de Barcelona, Bellaterra, Spain; 3 Joint Research Unit CTFC—AGROTECNIO, Lleida, Spain; 4 Department of Crop and Forest Sciences, University of Lleida, Lleida, Spain; 5 Institute of Anthropology, University of Mainz, Mainz, Germany; 6 Center of Natural and Cultural Human History, Danube Private University, Krems, Austria; 7 Integrative Prehistory and Archaeological Science, University of Basel, Basel, Switzerland; University at Buffalo - The State University of New York, UNITED STATES

## Abstract

The El Argar society of the Bronze Age in the southeast of the Iberian Peninsula (2200–1550 cal BCE) was among the first complex societies in Europe. Its economy was based on cereal cultivation and metallurgy, it was organized hierarchically, and successively expanded its territory. Most of the monumentally fortified settlements lay on steeply sloped mountains, separated by fertile plains, and allowed optimal control of the area. Here, we explore El Argar human diets, animal husbandry strategies, and food webs using stable carbon and nitrogen isotope analysis of charred cereal grains as well as human and animal bone collagen. The sample comprised 75 human individuals from the sites of La Bastida (n = 52) and Gatas (n = 23), 32 domesticated and wild animals as well as 76 barley and 29 wheat grains from two chronological phases of a total time span of *ca*. 650 years. The grains indicate extensive cereal cultivation under rain-fed conditions with little to moderate application of manure. Especially at La Bastida, crops and their by-products contributed significantly to the forage of the domesticated animals, which attests to a strong interrelation of cultivation and animal husbandry. Trophic level spacing and Bayesian modelling confirm that human diets were largely based on barley with some contribution of meat or dairy products. A cross-sectional analysis of bone collagen suggests that children were breastfed until about 1.5–2 years old, and infants from Gatas may have suffered from more metabolic stress than those at La Bastida. Adults of both sexes consumed similar diets that reflect social and chronological variation to some extent. Despite significantly higher δ^13^C and δ^15^N values at La Bastida than at Gatas, the isotopic data of the staple crops and domestic animals from both sites indicate that such differences do not necessarily correspond to different average human diets, but to agricultural strategies. These results urge for a reassessment of previous isotope studies in which only human remains have been taken into account. The study highlights that disentangling the complex influences on human isotope compositions requires a firm set of comparative data.

## Introduction

The relationship between *political centralisation* and *social inequality* has been a strongly debated topic during the last years, not only in archaeology and history, but also in economic and sociological studies [1–3]. All these disciplines debate the origins and consequences of *social inequality* and strive for adequate methods to quantify it.

The Bronze Age archaeological culture of El Argar (2200–1550 BCE) on the south-eastern Iberian Peninsula represents an outstanding example of an early complex society in European prehistory with ample evidence for social stratification [4]. Fortified hilltop settlements were home to large population sizes. Extensive storage facilities document that the cultivation of barley and wheat was among the key economic factors that secured long-term sustainability of the population, even though the steep slopes of the hills and their near surroundings offered poor conditions for agricultural production in what is today one of the driest regions in Europe (http://www.fao.org/nr/water/aquamaps/). Successful agricultural production in the valleys and plains as well as the redistribution of the harvests to the hilltop settlements implies the existence of strong rulers. Moreover, the archaeological record of the funerary remains attests to pronounced social inequality.

These circumstances of environmental imponderability and evidence for extensive accumulation of wealth raise fundamental questions regarding food production and the reflection of social stratification in average human dietary compositions. Bioarchaeological investigations can contribute to these discussions by carrying out stable isotope analyses–mainly of carbon (δ^13^C) and nitrogen isotopic composition (δ^15^N)–on plant, animal, and human remains [5–7]. Here we present δ^13^C and δ^15^N data of wheat and barley grains as well as bone collagen of domestic animal and human remains from the El Argar sites of La Bastida and Gatas in southeastern Iberia. The samples represent different positions of the trophic chain including producers and consumers. The study aims on revealing insights into food production strategies, the possible application of irrigation and manuring, as well as detecting the importance of plants, animals and marine resources in the human diet. Numerous factors influence the stable isotope composition of bone collagen and only a part of them is directly related to the proportions of certain foodstuffs. Conversely, many parameters that characterize dietary quality do not influence the isotope signals. For example, different kinds of meat from the same animal or ways of cooking and food preparation determine the taste and value of food, but can hardly be discerned by isotope analysis. A major objective of this study is therefore to explore and untangle the complex interrelation of isotopic signals of potential producers and consumers along food chains in an environmentally and socially complex archaeological setting.

In addition to the general characterization of human diets, we explore the data for interdependencies of dietary habits during lifetime and internal differentiation of the burial communities regarding sex, age, and the reflection of social status in the archaeological record [8]. Because both sites revealed numerous skeletal remains of subadult individuals, the exploration of weaning ages and possible metabolic stress in early childhood received specific attention in data evaluation.

## Archaeological context

**Fig 1:**
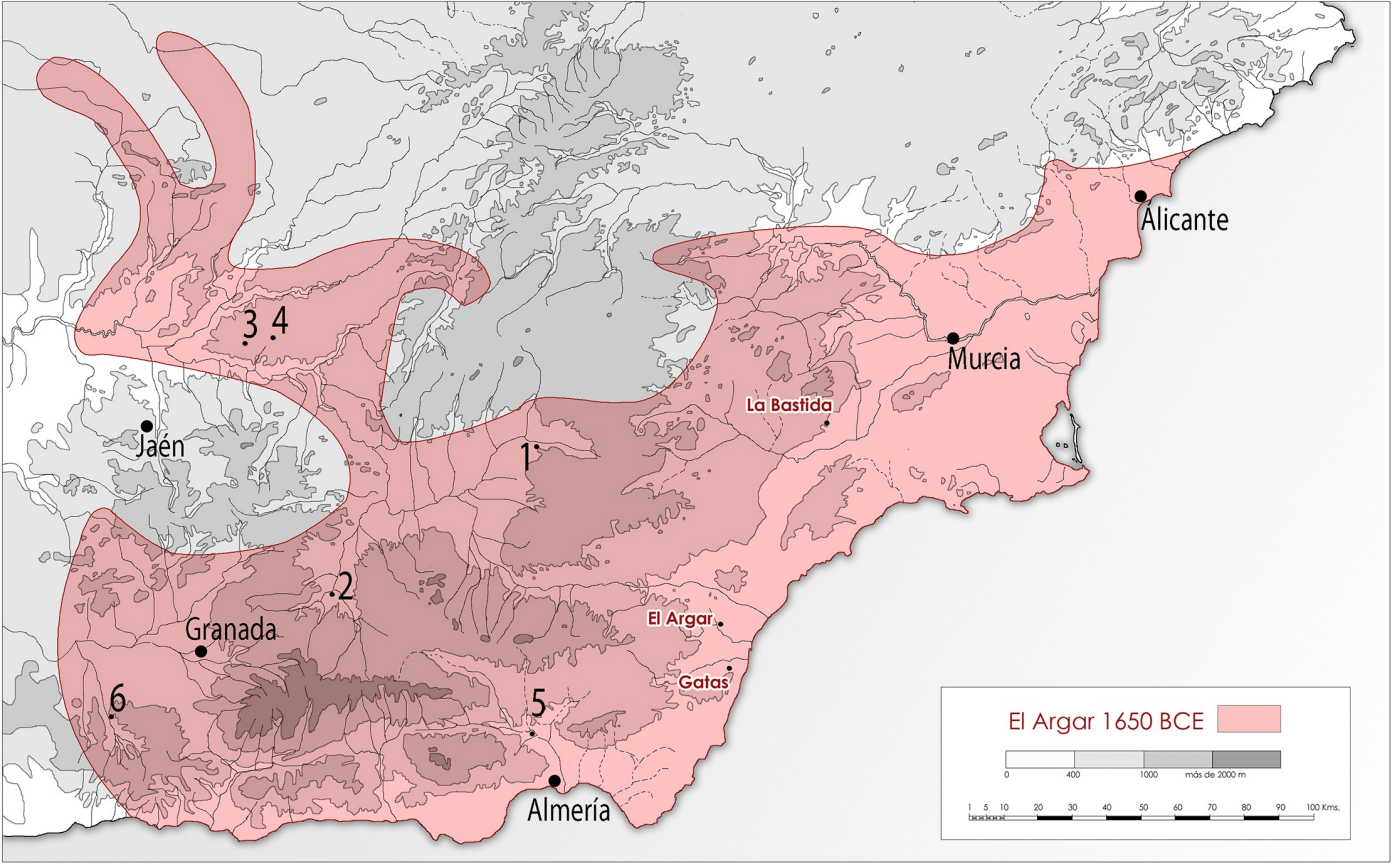
Maximum territorial extension of the El Argar culture and locations of the analysed sites of La Bastida and Gatas. Other sites with isotopic analyses of human remains: 1. Cerro de la Virgen, 2. Cuesta del Negro, 3. Baeza, 4. Úbeda, 5. Los Millares, 6. La Navilla (©ASOME, UAB).

El Argar emerged around 2200 BCE, after the destruction and abandonment of most Copper Age settlements, in a series of interconnected plains of north-eastern Almería and southern Murcia (southeast Iberian Peninsula), occupying initially an area of *ca*. 2500 km^2^ [4, 9] (Fig 1).

The recent excavations at La Bastida with its monumental fortification walls [10] suggest that El Argar was a highly complex political system based on economic specialisation and the exploitation of large territories. Most Argaric settlements were located on steep hills at the foot of mountain ranges, in many cases detached from the fertile plains but with optimal visual control over these areas and their communication routes [11]. All the Argaric settlements shared the same intramural funerary ritual. After *ca*. 1950 BCE a growing part of the population, particularly children, was included in the Argaric burial rite. Distinctive sets of funerary offerings differentiated the dead regarding their sex and age and revealed five relatively standardised categories of value, which seem to correspond to at least three social classes [12]. These differences can be observed from childhood onwards, which suggests that social position and access to economic means of production and political power were inherited [13]. While elite burials with specific metal weapons and ornaments represent about 10% of the funerary record, another 50% of the burials can be related to a class with political rights whose members were buried with common metal tools, weapons, ornaments, as well as ceramic vessels. A lowest sector comprised individuals with very modest grave-goods or no offerings at all and seems to represent some type of servants or slaves [4, 14]. The economic organisation of the hilltop settlements with specialised workshops and storage spaces, in which large working teams processed food and other resources, also points to the existence of an exploited class [15]. Different archaeological features suggest that El Argar developed around 1750 BCE into a state society based on a tributary system controlled by a dominant class through the central hilltop settlements [4, 16, 17]. At that time, it occupied the whole of southeast Iberia and the southern parts of the central Spanish Meseta, an area of approximately 35,000 km^2^. Most of the settlements were abandoned or destroyed around 1550 BCE, apparently due to social conflicts rising from subsistence shortages, caused by unsustainable agricultural practices [18].

**Fig 2:**
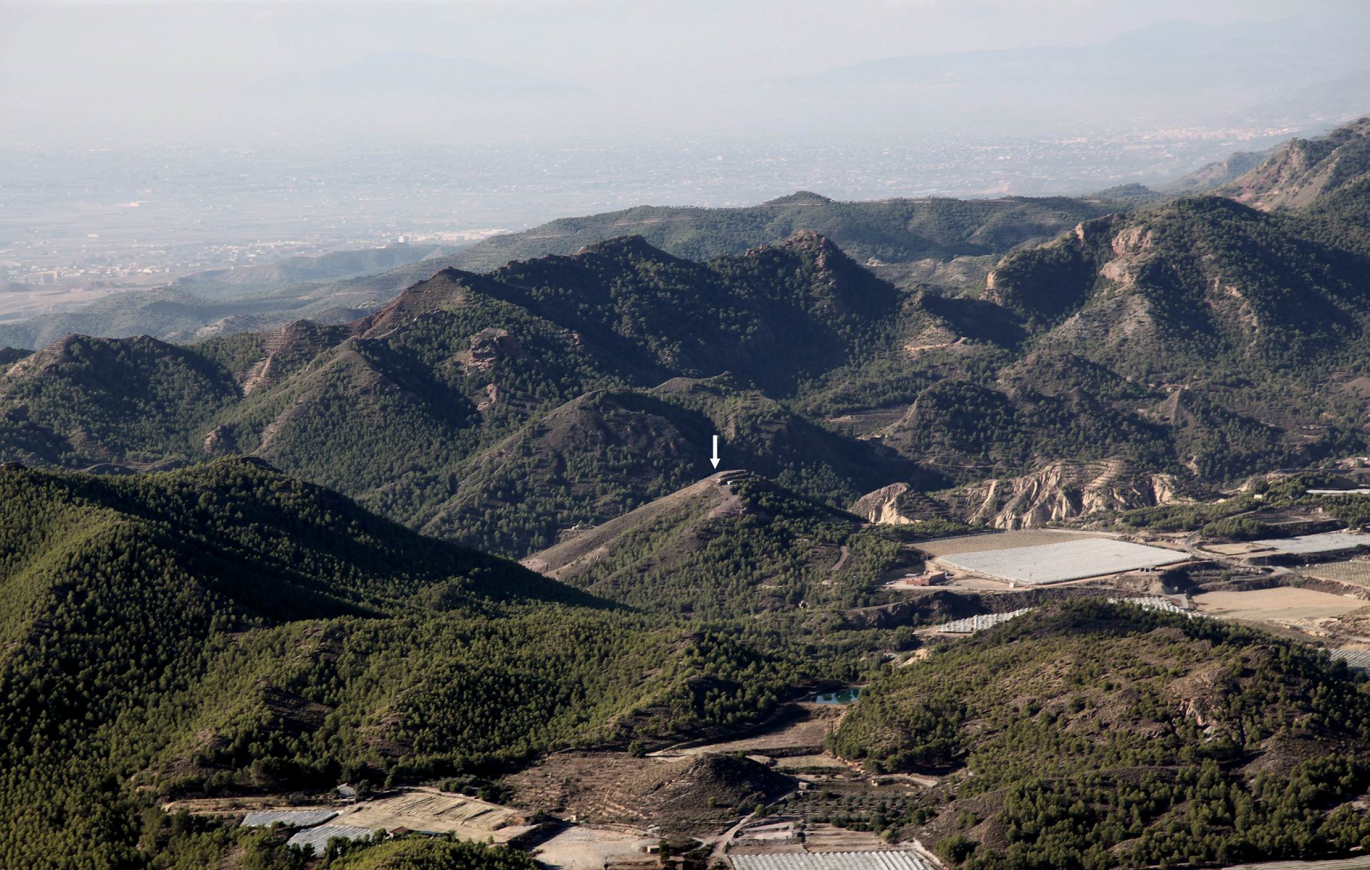
Location of the site of La Bastida between the mountain ranges of Espuña and La Tercia. The arrow marks the summit of the hilltop settlement. The fertile valley of the Guadalentín can be seen in the background (©ASOME, UAB).

This study focuses on botanical, faunal and skeletal remains from Gatas (Turre, Almería) and La Bastida (Totana, Murcia), two Argaric hilltop settlements with clear social and economic differences in the productive as well as the funerary realm [10, 19]. Ample series of ^14^C dates on short-lived material from funerary and domestic contexts have confirmed that the Argaric occupation of both sites lasted between *ca*. 2200 and 1550 BCE, whereas the majority of the funerary structures can be dated after 1950 BCE. A middle (phase 2) and a late phase (phase 3) dated respectively between *ca*. 2000–1750 BCE and *ca*. 1750–1550 BCE. Both settlements occupied hills that were naturally defended by vertical slopes, in a mountainous topography shaped by Alpine orogeny (Fig 2).

Though husbandry, particularly of sheep and goat, could have been carried out on the slopes surrounding the settlements, the main agricultural territories must have lain in the fertile valleys of the Guadalentín and the Aguas rivers, as it has been confirmed by GIS spatial modelling [20]. The exceptional quantities of grinding tools stand in stark contrast to the scarcity of sickle elements and imply that most of the population of the hilltop settlements was not directly involved in agricultural production [21]. The basic subsistence rather seems to have been supplied by communities living in the fertile lowlands, where dispersed, small-scale settlement remains have been identified. Today, the mean annual rainfall in both Tertiary basins is 273 mm at La Bastida and 250 mm at Gatas, although differences between the driest and the wettest years can reach a factor of 13. While barley can be sown and harvested during most of the years, wheat production is very difficult without irrigation. Paleoclimatic proxies suggest that both temperature and rainfall were slightly higher than today in the area at the end of the 3^rd^ and the beginning of the 2^nd^ millennium BCE [18, 22–24].

## Nitrogen and carbon isotope analysis

Stable nitrogen and carbon isotope analysis of bone collagen is a well-established method for past dietary reconstruction [25–27]. Dietary protein is the primary source of nitrogen in collagen, whereas carbohydrates and fats do not contain any nitrogen. Most of the carbon in collagen, which is itself a protein, also derives from proteins, while a minor part of the carbon originates from carbohydrates and fats [28]. Isotope fractionation causes variation of the isotopic compositions of foodstuffs and along trophic chains. The major source of distinction among carbon isotope compositions in terrestrial domains is the photosynthetic pathway of the plants at the base of the foodwebs. C_3_ plants, which prevail on the Iberian Peninsula, have in general lower carbon isotope values (δ^13^C = -35 to -22‰ V-PDB) than C_4_ plants (δ^13^C = -12.7 to -11.4‰) [29]. Among the latter, millet is the most relevant staple crop in Prehistoric Europe [30, 31], but it is unattested in El Argar. Isotope fractionation during metabolic processes and collagen formation raises the δ^13^C values of herbivore collagen by about 5‰ in comparison to the ingested plants [28, 32], whereas the difference of collagen δ^13^C between representatives of two adjacent trophic levels is 0.8 to 1.3‰ [33–35]. The enrichment of heavy stable isotopes with increasing trophic levels also causes considerable variation among nitrogen isotope values (δ^15^N), which increase by about 3 to 5‰ [36] or even 6‰ per trophic level [37]. The relative accumulation of the heavy isotopes along food chains implies that higher δ^15^N values of human collagen indicate higher proportions of animal-derived foodstuffs, such as meat and dairy products. In agricultural societies, δ^15^N values are therefore often considered especially informative regarding the possible reflection of social differentiation as manifested in the access to more valuable foodstuffs. The explicit or implicit assumption is that the production of foodstuffs from organisms of a higher trophic level, such as meat or dairy products, requires a larger labour input. They are, therefore, considered to be more costly than plant-based foodstuffs, such as cereals or pulses [8, 38–41].

Moreover, aquatic resources may have had relevant influence on the isotope composition of the human collagen, even though their isotope values are often highly variable [42]. In general, marine fish and mammals contribute dietary protein with relatively high δ^13^C and δ^15^N values [42, 43] with average values of -13 to -10‰ (δ^13^C) and 7 to 11‰ (δ^15^N) in ancient Mediterranean samples [44]). Freshwater fish tend to have similar or lower δ^13^C values than terrestrial mammals in C_3_ habitats (global estimates, incl. Iberia: *ca*. -35 and -25 ‰) and similar or higher δ^15^N values (*ca*. 8–16 ‰) [45–47].

While dietary reconstruction based on isotope fractionation appears to be a plausible principle, environmental and anthropogenic factors may obscure straightforward interpretations. Regarding the δ^13^C values of C_3_ plants at the base of foodwebs, variation occurs depending on vapour pressure deficit, solar irradiance, or soil humidity [48–50]. Drought raises the δ^13^C values of rain-fed crops so that charred cereal grains can indicate water status or provide indirect evidence for irrigation [51–53]. The δ^13^C values of the aerial CO_2_ also varied over time and decreased markedly after the 1850s due to the Suess effect [54]. To account for this effect, carbon isotope discrimination (Δ^13^C) can be used for the comparison of carbon isotope compositions of plant data independent of their chronological placement [54, 55].

Regarding the nitrogen isotope values, the application of animal manure to agricultural fields causes remarkable variation among staple crops [56–58]. The increase of the δ^15^N values of the grains can bias estimations regarding the relative contributions of plant and animal-derived foodstuffs. Moreover, charring–the prerequisite of preservation of cereal grains from non-waterlogged conditions–may alter the original isotopic compositions of plant tissues [59, 60].

Carbon and nitrogen isotope data are often explored regarding breastfeeding and weaning. The latter starts with the introduction of complementary foods and ends with the complete cessation of breastmilk consumption. Metabolic processes cause isotope fractionation, due to which breastmilk is enriched in the heavy ^15^N and ^13^C isotopes in compared to the mother’s diet [61–63]. Analyses of hair samples documented about 0.9‰ higher δ^15^N and 0.4‰ higher δ^13^C values in new-born infants compared to their mothers [64]. The differences between isotope compositions of the same kind of tissue increase to 2–3‰ higher δ^15^N and 1‰ higher δ^13^C values in samples of the infants during the breastfeeding period [65–67]. Nursing signals may overlap with the effects of metabolic stress during which bodily proteins are catabolized leading to higher δ^15^N values, whereas the breakdown of fats may contribute carbon that is depleted in ^13^C compared to protein-bound carbon [68]. The present study considers data of differently aged children for cross-sectional information [69]. This approach has caveats because it combines information from individuals with possibly varying personal histories of prolonged or shortened periods of breastfeeding and health conditions that led to a premature death. The latter may have been related to or have influenced dietary provision. Furthermore, temporal resolution of the isotopic signal may be poor due to variation in bone turnover and stunting [67]. A better approach to studying breastfeeding and weaning is serial sampling of tooth dentine, which reveals individualized information from persons who survived the critical period of breastfeeding and weaning [67, 70, 71]. However, because this project focussed on the general characterisation of early Bronze Age diets, we explored the collagen data of the bones of the infants and did not conduct any serial sampling of teeth.

## Materials

This study investigated the carbon (δ^13^C) and nitrogen isotope composition (δ^15^N) of charred cereal grains as well as of animal and human bone collagen from two hilltop settlements of the El Argar society in southeastern Spain. The selected samples included 75 human individuals (52 from La Bastida and 23 from Gatas), which represent the middle and late phases of the El Argar period [14].

All samples from La Bastida have been recovered during excavations conducted by the Universitat Autònoma de Barcelona (UAB), except for BA 04, which belongs to tumb 4 excavated by the Siret brothers in the 19^th^ century. The skeletons from the modern excavations of La Bastida are kept at the Archaeological Research Centre of La Bastida, Totana, Spain, were they are inventoried according to their grave numbers. Remains of burial BA-04 are kept at the MRAH of Brussels, Belgium, and were sampled with the permission of Dr. Nicolas Cauwe, the custodian responsible for the El Argar collection at the MRAH. All samples from Gatas are kept at the Archaeological Laboratory ASOME of the UAB, Barcelona, Spain, and are registered according to their grave numbers.

Six samples were excluded from data interpretation due to insufficient collagen preservation [72]. The 48 individuals from La Bastida that yielded evaluable results comprised 23 infans I (0 to 6 years of age at death), three infans II (7 to 13 years), two juveniles (14 to 20 years) and 20 adults (> 21 years). Among the adults were nine males/probably males and 11 females/probably females (S1A Table). The sample from Gatas was composed of 21 individuals with high quality collagen, among them nine infans I, one infans II, two juveniles, and nine adults with a nearly balanced sex ratio of five females and four males. Samples were preferentially taken from the ribs. Especially at Gatas, other skeletal elements were also selected due better bone preservation.

Additionally, 28 bones of domesticated animals (*Bos taurus*, *Sus domesticus*, *Ovis aries*, *Capra hircus*) and four deer bones (*Cervus elaphus*) served as comparative samples. Three samples of domesticated animals from La Bastida failed the quality criteria of good collagen preservation. The faunal dataset for further evaluation included 17 bones from La Bastida and 12 bones from Gatas (S1B Table). The animal bones from La Bastida are kept at the Archaeological Research Centre of La Bastida, Totana, Murcia, and those from Gatas are kept at the Archaeozoological Laboratory of the UAB, Barcelona, Spain.

Furthermore, charred grains of hulled barley (*Hordeum vulgare* L.) and naked wheat (*Triticum aestivum*/*durum*, after [73]) were sampled from both sites. They were collected from floor levels and storage jars inside housing structures in the settlement area. To recover plant remains, soil samples were treated using a standard flotation tank with 5-, 2- and 0.5-mm sieves. In total, 76 barley grains (66 from La Bastida and 10 from Gatas) and 29 wheat grains (22 from La Bastida and 7 from Gatas) were analysed (S1C Table). They covered a temporal range of approximately 650 years (dated from 2200 to 1550 cal. BCE).

## Analytical methods

Collagen extraction for human and animal samples followed [74] with some modifications [75]. The bone surfaces were manually removed and cleaned with dental cutting and milling equipment. Bone samples were demineralized in 10 ml of 0.5 N HCL for about 14 days. After neutralization with deionized water, the samples were treated with 0.1 M NaOH for 24 h to dissolve humic acids and rinsed again to neutrality. Gelatinization was conducted at 70°C for 48 h. The collagen was then filtered with Ezee-Filter^™^ separators (Elkay) and concentrated using Amicon© ultrafilters (Millipore; cutoff, <30 kDa). The purified collagen was frozen and lyophilized for 48 h. One to two milligrams of dried collagen were weighed into tin foil capsules. The δ^13^C (‰ vs. V-PDB) and δ^15^N (‰ vs. AIR) values were determined in duplicates by an elemental analyser (vario EL III, Elementar Analytical Systems) and the isotope ratios were measured by an IsoPrime High Performance isotope ratio mass spectrometer (IRMS; VG Instruments). The data were corrected using two-point calibrations based on USGS 40 and IAEA N2 for nitrogen and CH6 and CH7 for carbon. Measurement errors were smaller than ± 0.2‰ for δ^15^N and ± 0.1‰ for δ^13^C (1 SD). Treatment and measurement of human and animal samples for carbon and nitrogen stable isotopes were carried out in the laboratories of the Institute of Geosciences (Department of Applied Palaeontology) and the Institute of Organic Chemistry at the University of Mainz (Germany).

Charred grains were soaked separately with 6 M HCl for 24 h at room temperature to remove carbonate crusts [76], which is a crucial step to avoid δ^13^C shifts, and then rinsed repeatedly with distilled water. Then, they were oven dried and milled separately at the Department of Crop and Forest Sciences of the University of Lleida (Spain). For the determination of δ^13^C and δ^15^N an aliquot of each sample was weighed into tin foil capsules and combusted using an elemental analyser interfaced to an isotope ratio mass spectrometer at the UC Davis Stable Isotope Facility (California, USA). The accuracy of analyses was 0.06‰ for δ^13^C and 0.2‰ and for δ^15^N.

In order to account for isotopic shifts due to charring, we subtracted 0.31‰ from the δ^15^N values of the charred cereal grains [60, 77] and did not correct for the minimal and unsystematic influence of charring on the δ^13^C values [78]. Carbon isotope discrimination (Δ^13^C) was calculated according to [55] with a correction factor of -6.39‰ as interpolated for 2200–1550 cal. BCE [54]. In dietary reconstructions, we considered an increase of 4.8‰ between the δ^13^C values of the plants and the collagen of their consumers and 0.8‰ between the carbon isotope values of the collagen of representatives of the previous trophic level [77]. For nitrogen isotope compositions, we used a diet-consumer-offset of 4.0‰ in our considerations about dietary compositions [77].

For data evaluation and statistical analysis, Microsoft Excel, IBM SPSS Statistics software, Version 19 for Windows (SPSS Inc.), and SigmaPlot (version 14.0) were used. Bayesian modelling of dietary compositions was performed using the FRUITS software [7].

## Results

### The human samples

**Fig 3:**
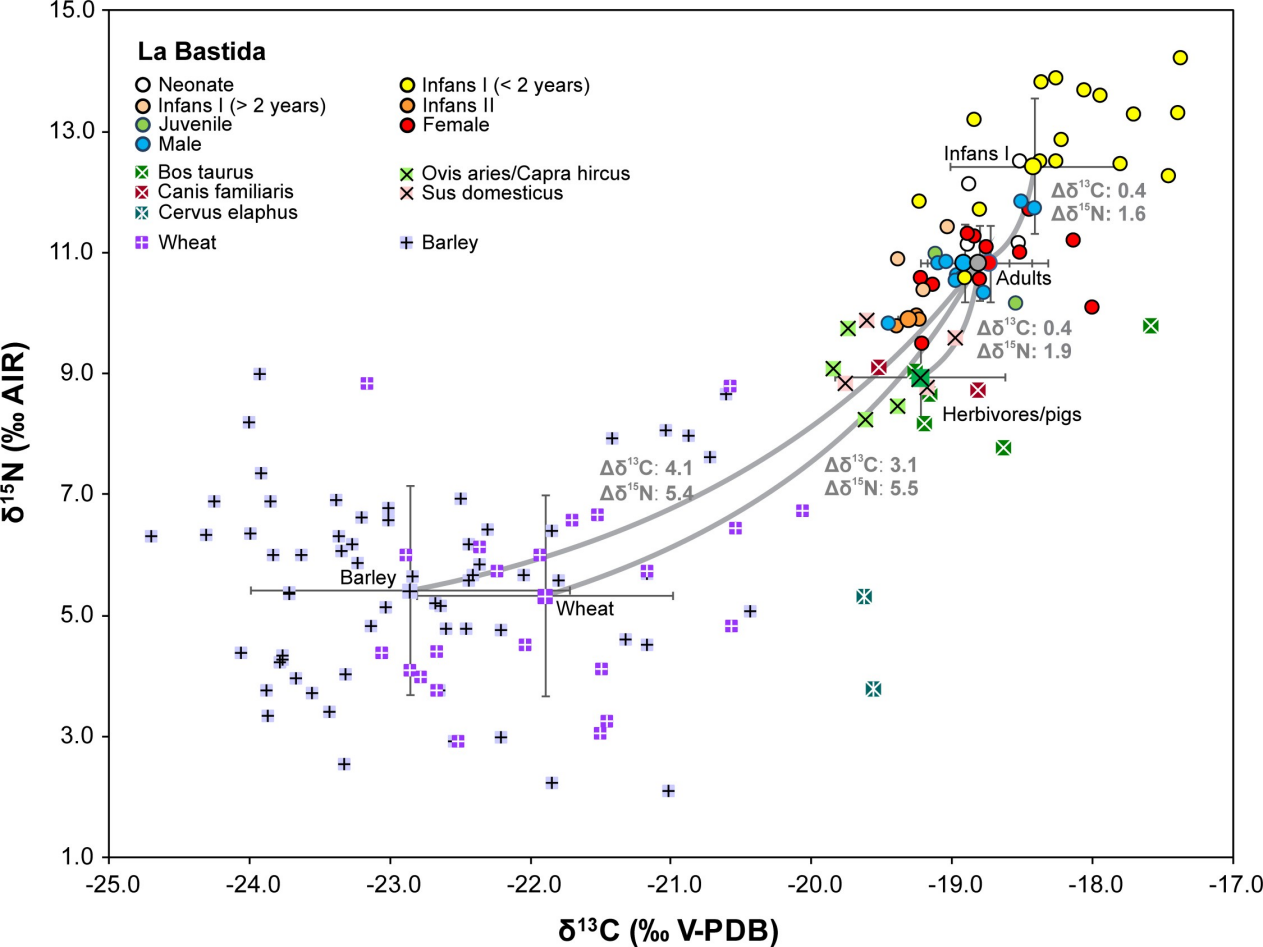
Carbon and nitrogen isotope compositions of charred cereal grains as well as animal and human collagen from the site of La Bastida. Larger symbols mark the averages of different data groups with one standard deviation. The grey lines link members of adjacent trophic levels and give their mean isotopic offsets.

Stable isotope values of the humans from the site of La Bastida (n = 48) ranged from −19.5‰ to −17.4‰ (mean = −18.6 ± 0.5‰) for δ^13^C and from 9.5‰ to 14.2‰ (mean = 11.5 ± 1.3‰) for δ^15^N (S1A Table; S1D Table; Fig 3).

**Fig 4:**
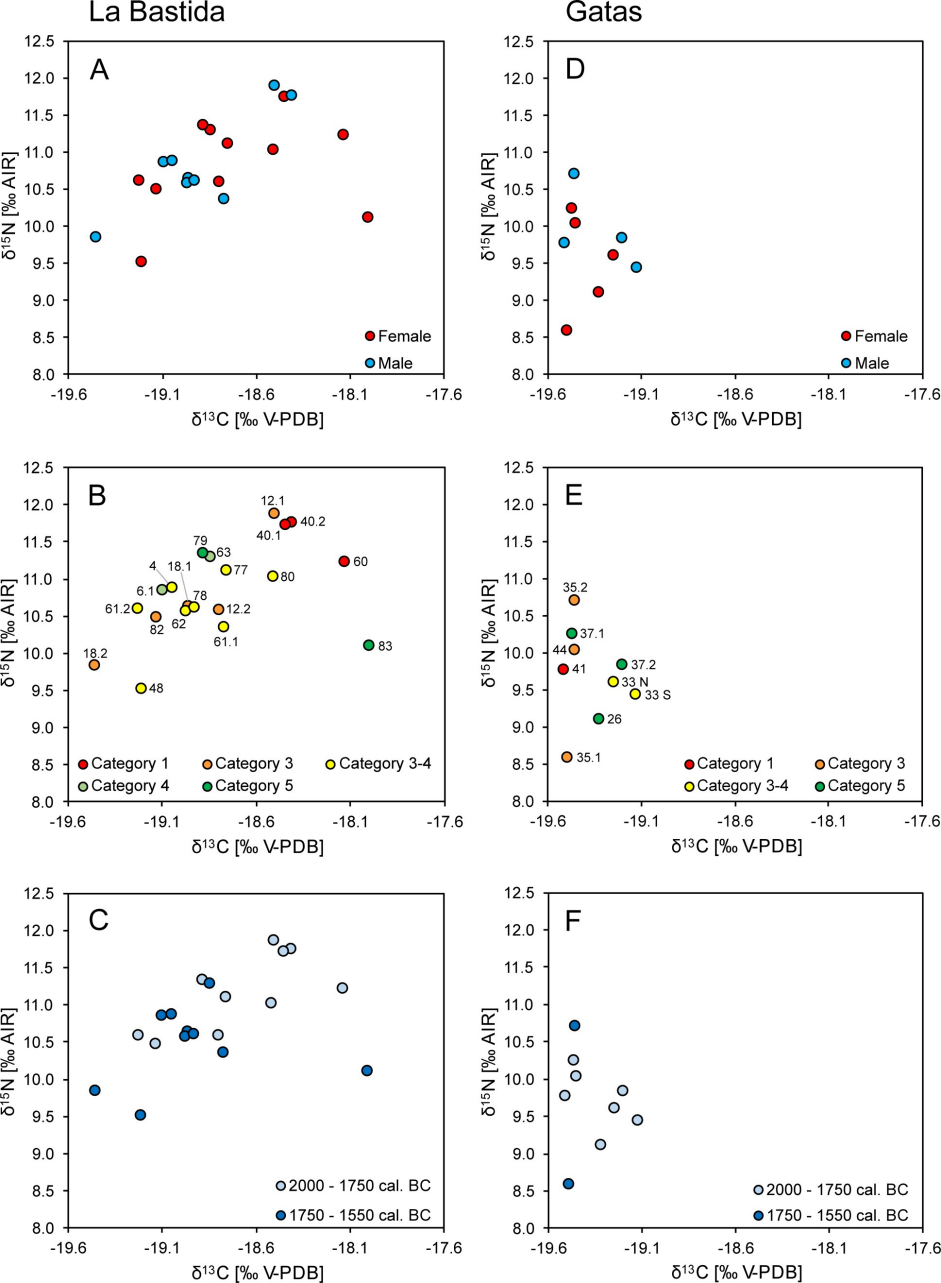
Internal differentiation of carbon and nitrogen isotope compositions of the adult individuals from La Bastida (left) and Gatas (right) according to sex (A and D), social categories (B and E), and chronological affiliation (C and F). The individuals are labelled in graphs B and E.

The difference between the infants and adults determined the internal variation of the dataset. Both the δ^13^C (mean: -18.4 ± 0.6‰) and the δ^15^N values (mean: 12.4 ± 1.1‰) of the children of the age group infans I (n = 23) were higher than those of the older children (means: δ^13^C: -19.3 ± 0.1‰; δ^15^N: 9.9 ± 0.1; n = 3), juveniles, and adults (means: δ^13^C: -18.8 ± 0.4‰; δ^15^N: 10.8 ± 0.6; n = 20). Among the adults, the average δ^13^C and δ^15^N levels of males and females were almost identical (male means: δ^13^C: -18.9 ± 0.3‰; δ^15^N: 10.8 ± 0.6; n = 9; female means: δ^13^C: -18.7 ± 0.4‰; δ^15^N: 10.8 ± 0.6; n = 11). Differences between the sexes were non-significant (Student’s *t*-test: δ^13^C: t(18) = -1.090, *p* = 0.290; δ^15^N: t(18) = 0.001, *p* = 0.999) (Fig 4 A; S1D Table).

Regarding social differentiation, the three individuals assigned to the top category [12] were among those with the highest δ^13^C (-18.4 to -18.1‰) and δ^15^N values (11.2 to 11.7‰) (Fig 4B). Remarkably, the data for the male and the female in the double inhumation of grave 40 were almost identical. One individual from the next lower represented social category 3 (BA 12/1) had very similar, high isotope values to both burials in grave 40. The isotope data of the representative of the other social categories (3 to 5) scattered in similar ranges. The average δ^15^N values varied over time (Fig 4C). The difference between the mean values of 11.2 ± 0.5‰ in the middle phase 2 (2000–1750 cal. BCE) and 10.5 ± 0.5‰ in the later phase 3 (1750–1550 cal. BCE) was statistically significant (Student’s *t*-test: t(18) = 3.065, *p* = 0.007). However, it should be kept in mind that no upper class individuals (with expectedly high δ^15^N values) dating to the phase 3 have been analysed. Thus, the observed chronological variation may have been affected by incomplete sampling. The average δ^13^C values remained very similar in both chronological phases (mean phase 2: -18.7 ± 0.3‰; phase 3: -18.9 ± 0.4‰).

**Fig 5:**
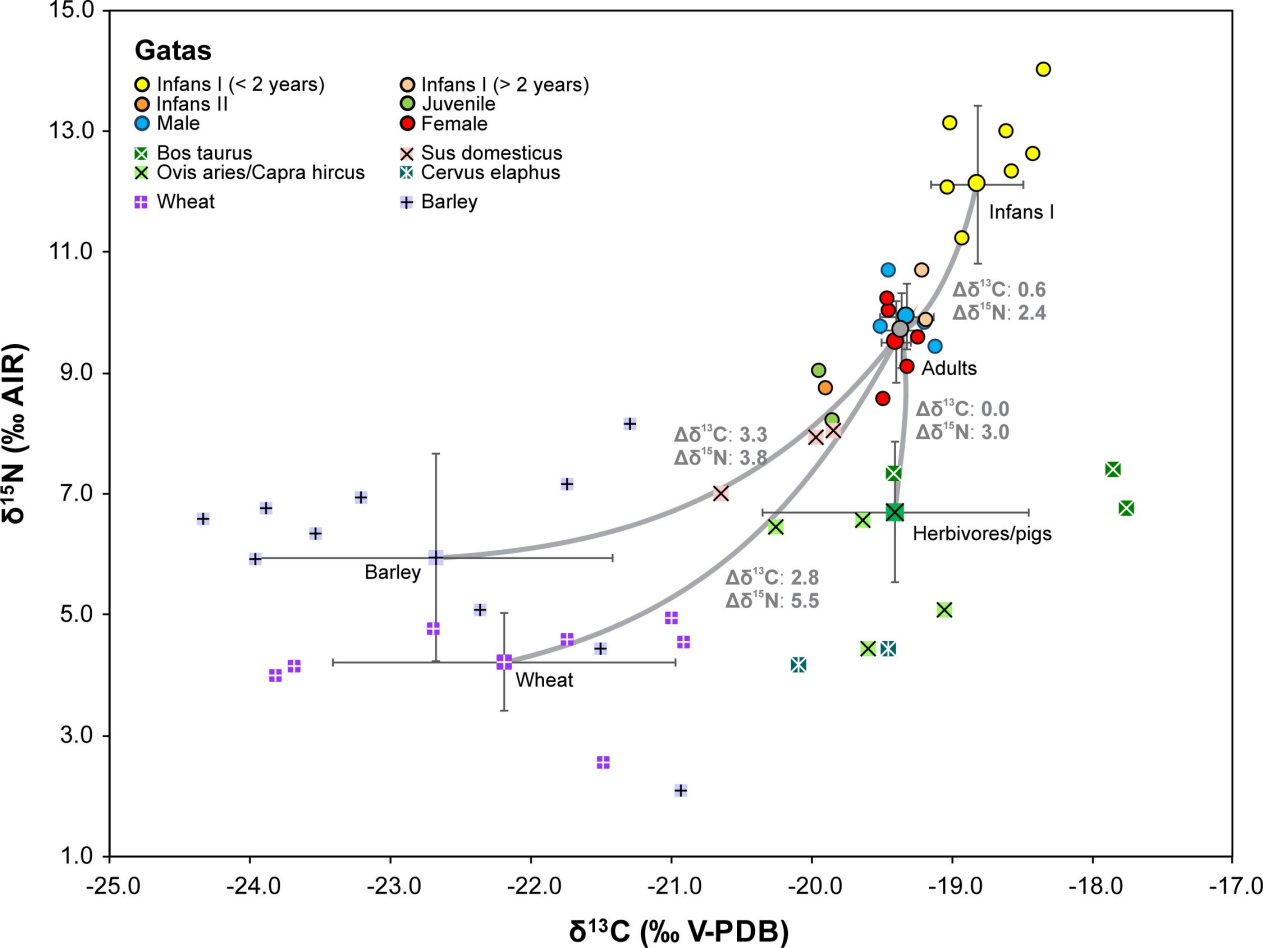
Carbon and nitrogen isotope compositions of charred cereal grains, animal and human collagen from the site of Gatas. Larger symbols mark the averages of different data groups with one standard deviation. The grey lines link members of adjacent trophic levels and give their mean isotopic offsets.

The twenty-one human individuals analysed from Gatas showed isotopic values ranging from -20.0‰ to -18.3‰ (mean = -19.2 ± 0.5‰) for δ^13^C and from 8.2‰ to 14.0‰ for δ^15^N (mean = 10.6 ± 1.7‰) (S1A Table; Fig 5).

**Fig 6:**
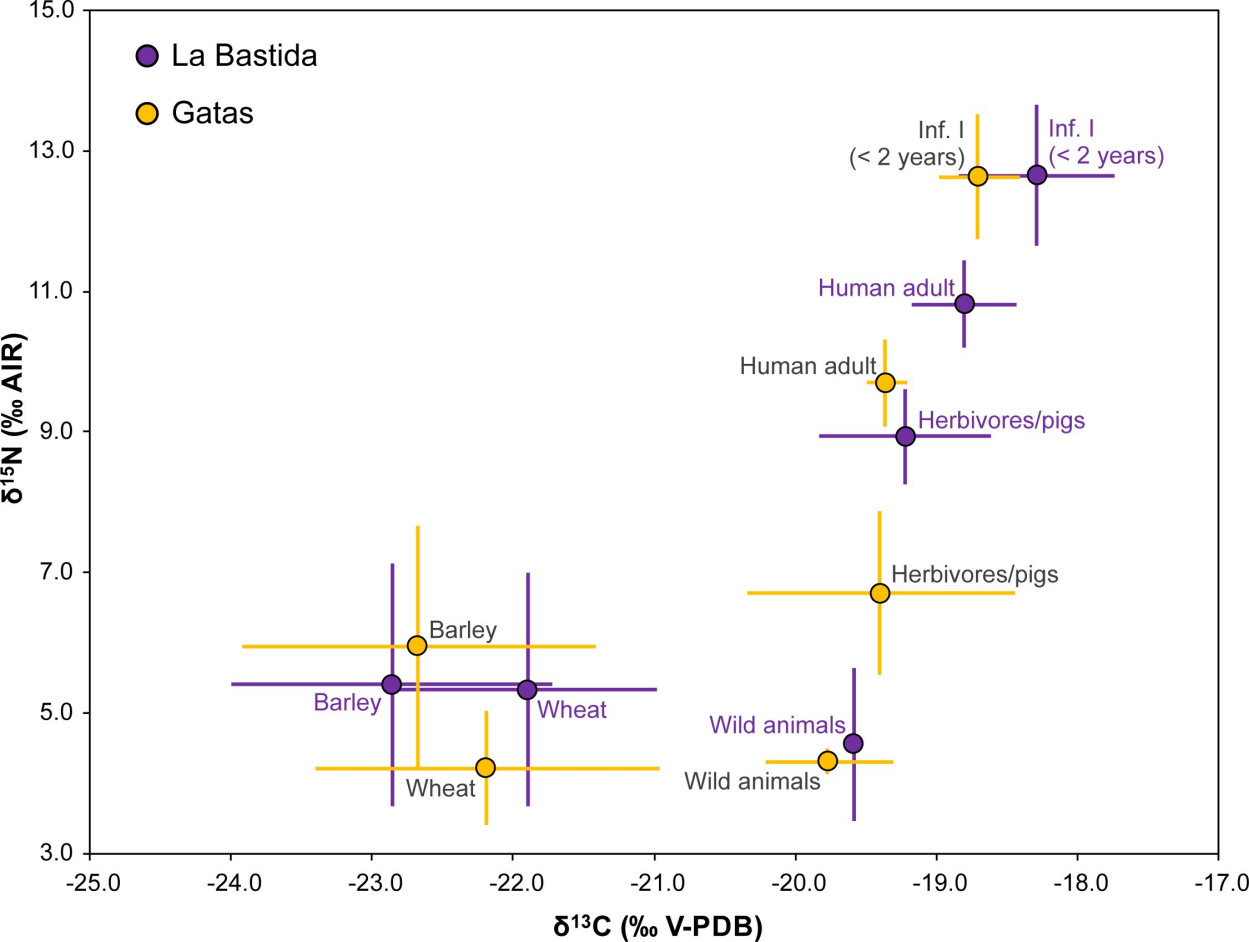
Averages and one standard deviation of the main data groups from La Bastida (purple) and Gatas (yellow).

As in La Bastida, most of the variation within the dataset was due to the high isotope values of the children of the age group infans I (mean δ^13^C: -18.8 ± 0.3‰; δ^15^N: 12.1 ± 1.3‰) in comparison to all older age categories (e.g. adult mean δ^13^C: -19.4 ± 0.1‰; δ^15^N: 9.7 ± 0.6‰). The isotope compositions of males and females were very similar (S1D Table; Fig 4D). The slightly higher average δ^15^N value of the males (9.9 ± 0.5‰) than of the females (9.5 ± 0.7‰) was basically caused by the male GA 35/2 with a high δ^15^N value and the female GA 35/1 with a low one. Similarly, the mean δ^13^C values were almost identical for males (-19.3 ± 0.2‰) and females (-19.4 ± 0.1‰). The differences between the sexes were non-significant (Student’s *t*-test: δ^13^C: t(7) = -0.738, *p* = 0.485; δ^15^N: t(7) = -1.010, *p* = 0.346). The sample sizes for the different social categories were low and did not reveal covariation with the isotope values (Fig 4E). The δ^13^C and δ^15^N values of burial GA 41, the only individual of the highest social category, were indistinguishable from the representatives of the other social categories. Of the two individuals of the younger chronological phase, one revealed the lowest and the other the highest δ^15^N values (Fig 4F). As tomb GA 35 was disturbed in Post-Argaric times and grave goods seem to have been extracted, it cannot be excluded that the comparatively high δ^15^N value of the male GA 35/2 distinguishes a member of the upper social categories of the final El Argar phase, as suggested by the presence of two silver spirals. In comparison to each other, both the δ^13^C and δ^15^N values of the adults from La Bastida are significantly higher and the δ^13^C are more variable than those from Gatas (Student’s *t*-test: δ^13^C: t(27) = 4.345, *p* ≤ 0.001 δ^15^N: t(27) = 4.471, *p* ≤ 0.001) (S1D Table; Fig 6).

### The faunal samples

The isotope data of the animal bone collagen are listed in S1B Table and summarized in S1D Table. At La Bastida, the δ^13^C values of the domestic animals ranged between -19.8 and -17.6‰ and the δ^15^N values between 7.8 and 9.9‰ (Fig 3). The domestic herbivores (cattle, sheep, goat) and pigs had remarkably higher δ^15^N values (mean: 8.9 ± 0.7‰) than the deer (3.8 and 5.3‰), while the δ^13^C values of the domestic animals (mean -19.2 ± 0.6‰) and the deer (both individuals: -19.6‰) overlapped. Among the domestic animals, the ranges of the δ^15^N values of cattle, sheep/goat and pigs were indistinguishable, while cattle tended to have higher δ^13^C values (mean: -18.8 ± 0.7‰) than sheep/goat and pigs (-19.9 ± 0.5‰). The isotope values of dogs fell among those of the domestic herbivores and pigs.

At Gatas, the δ^13^C values of the domestic animals ranged between -20.6 and -17.8‰ and the δ^15^N values between 4.4 and 8.1‰ (Fig 5). Again, deer had lower δ^15^N values (4.2 and 4.4‰) than the domestic animals (mean: 6.7 ± 1.2‰). However, the low δ^15^N values of a goat and a sheep (4.4 and 5.1‰) made the ranges of wild and domestic animals less distinct than observed at La Bastida. Among the domestic animals, pigs yielded higher δ^15^N values than sheep and goats, and cattle had higher δ^13^C values than the other domestic species.

Both the δ^13^C and δ^15^N values of the deer from La Bastida and Gatas were very similar to each other (Fig 6). This applies also to the δ^13^C values of the domestic herbivores and pigs from both sites. In contrast, the δ^15^N values of the domestic animals from La Bastida were on average more than 2‰ higher than the δ^15^N values of the domestic animals from Gatas. This difference was statistically significant (Student’s *t*-test: t(21) = 5.771, *p* ≤ 0.001). Moreover, the isotope compositions of the collagen samples of the domestic animals from Gatas were more variable, and the data ranges of the different species were more distinct than at La Bastida, even though this finding needs to be treated with caution due to the small sample sizes.

### The plant samples

The mean δ^13^C values of cereal grains ranged from -22.7‰ (barley in Gatas and La Bastida) to -21.8‰ (S1C Table; Fig 3; Fig 5). For Δ^13^C, these values varied between 14.0‰ (wheat in La Bastida) and 20.5‰ (barley in La Bastida), which are in all cases typical values of rainfed crops [54]. Wheat usually had higher δ^13^C values than barley, although this difference was marginally significant (Student’s *t*-test: t(86) = 1.872, *p* = 0.064) only for La Bastida. The observed differences in δ^13^C between wheat and barley can be attributed to the longer crop cycle of wheat, which forces the grain filling period to develop under drier conditions in spring. Likewise, the δ^15^N values of cereal grains ranged from 4.5‰ (wheat in Gatas) to 6.3‰ (barley in Gatas). There were significant differences (Student’s *t*-test: t(15) = 2.524, *p* = 0.023) in δ^15^N between wheat and barley in Gatas and marginally significant differences (Student’s *t*-test: t(27) = 1.853, *p* = 0.075) between La Bastida and Gatas for wheat (Fig 5). The larger δ^15^N records of barley as compared to wheat in Gatas, together with the higher values attained by wheat in La Bastida as compared to those of Gatas, suggest that the nutritional status of the wheat crop in Gatas was less optimal than in La Bastida, although still in the range of adequate levels of manuring [57].

## Discussion

### El Argar foodwebs

The long-term maintenance of a stable and successful society required a sustainable agrarian economy. The following considerations reflect on regional human-environment interrelations that allowed mastering the challenge of feeding the extensive human populations of the El Argar society in one of the driest regions in Europe.

### Growing conditions of cereals

**Fig 7:**
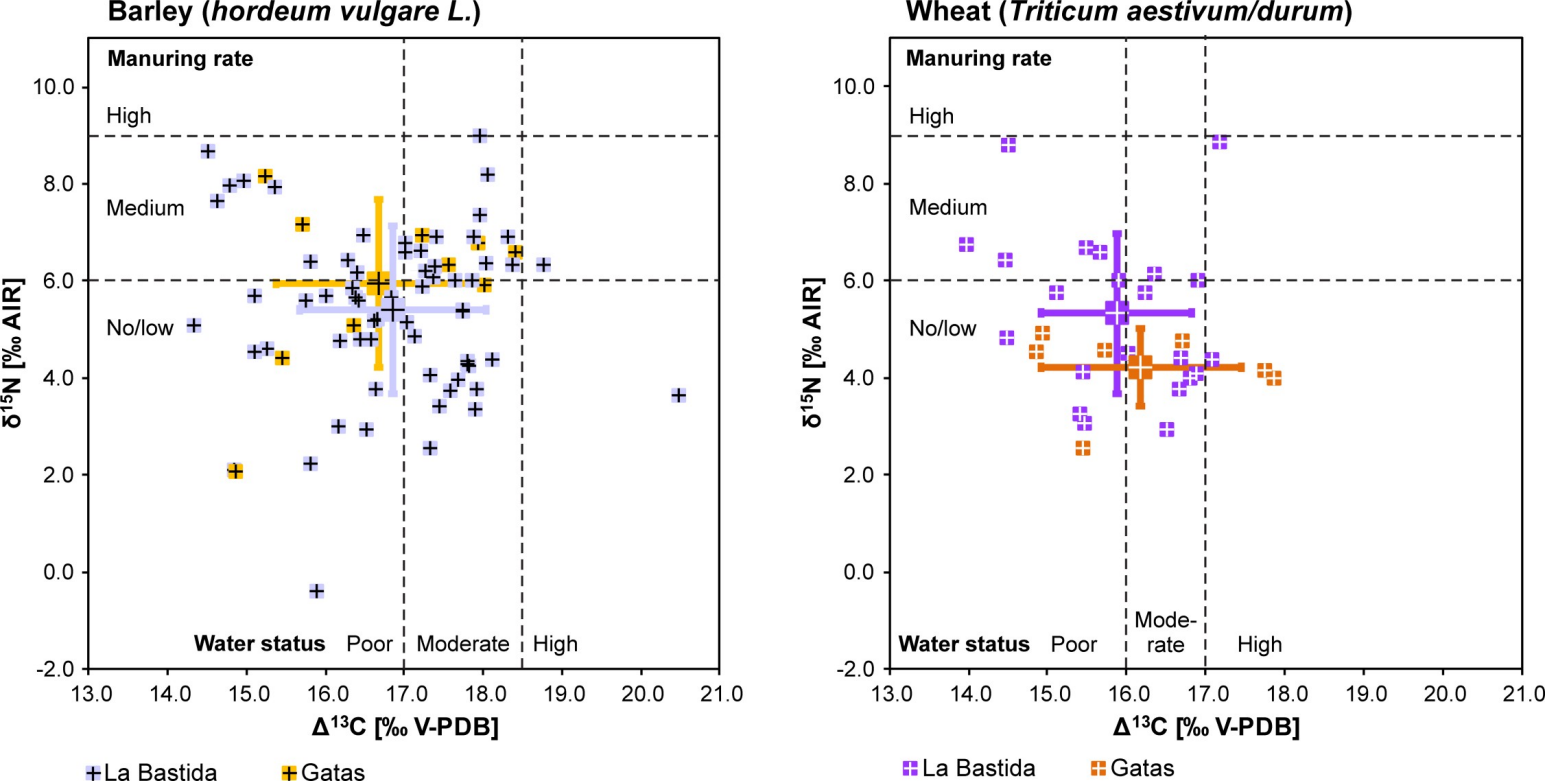
Carbon and nitrogen isotope data of barley (left) and wheat (right) from La Bastida (purple) and Gatas (yellow). The estimations of the water status are taken from [82, Fig 2]. Manuring rates are based on [80, Fig 4].

The overall dryness of the landscape in the growing period is reflected by the Δ^13^C values of the charred cereal grains, which are at the lower end of the data spectrum of C_3_ winter cereals [79]. The Δ^13^C values of the barley grains from La Bastida and Gatas (Fig 7) overlap with data from modern fields in Morocco that represent low precipitation rates [80] as well as with those from the Bronze Age site of Terlinques about 100 km NE of La Bastida and 200 km NE of Gatas [81].

Among the reasons for the relatively large range of Δ^13^C values are year-to-year variations of the amount of precipitation and the timing and duration of precipitation events. In addition, the data likely indicate origins in different catchment areas with varying natural moisture levels and micro-climate [51, 82]. The difference between the average Δ^13^C values of wheat and barley (about 1‰) is in the same range as observed in modern crops [54], and suggests that both kinds of crops were grown under overall similar conditions. The obtained data are in agreement with those from pioneering comprehensive work by Araus et al. [24, 53], that does not point to any deliberate irrigation, an interpretation which stands in contrast to that offered by Mora-González et al [81]. According to basic research by Wallace et al. and Styring et al. [51, 77, 80, 82], the carbon isotope values of the cereal grains from La Bastida and Gatas correspond to a wide range of poor to moderate water conditions, whereas deliberate irrigation should have resulted in higher and more uniform Δ^13^C values (Fig 7). Evidence for a poor water status in about half of the crops suggests that water availability was a limiting factor of growth in certain years or at certain locations. The isotopic values of seeds from different settlement phases do not show any significant differences and point towards an overall continuity of the agricultural practices at both sites.

**Fig 8:**
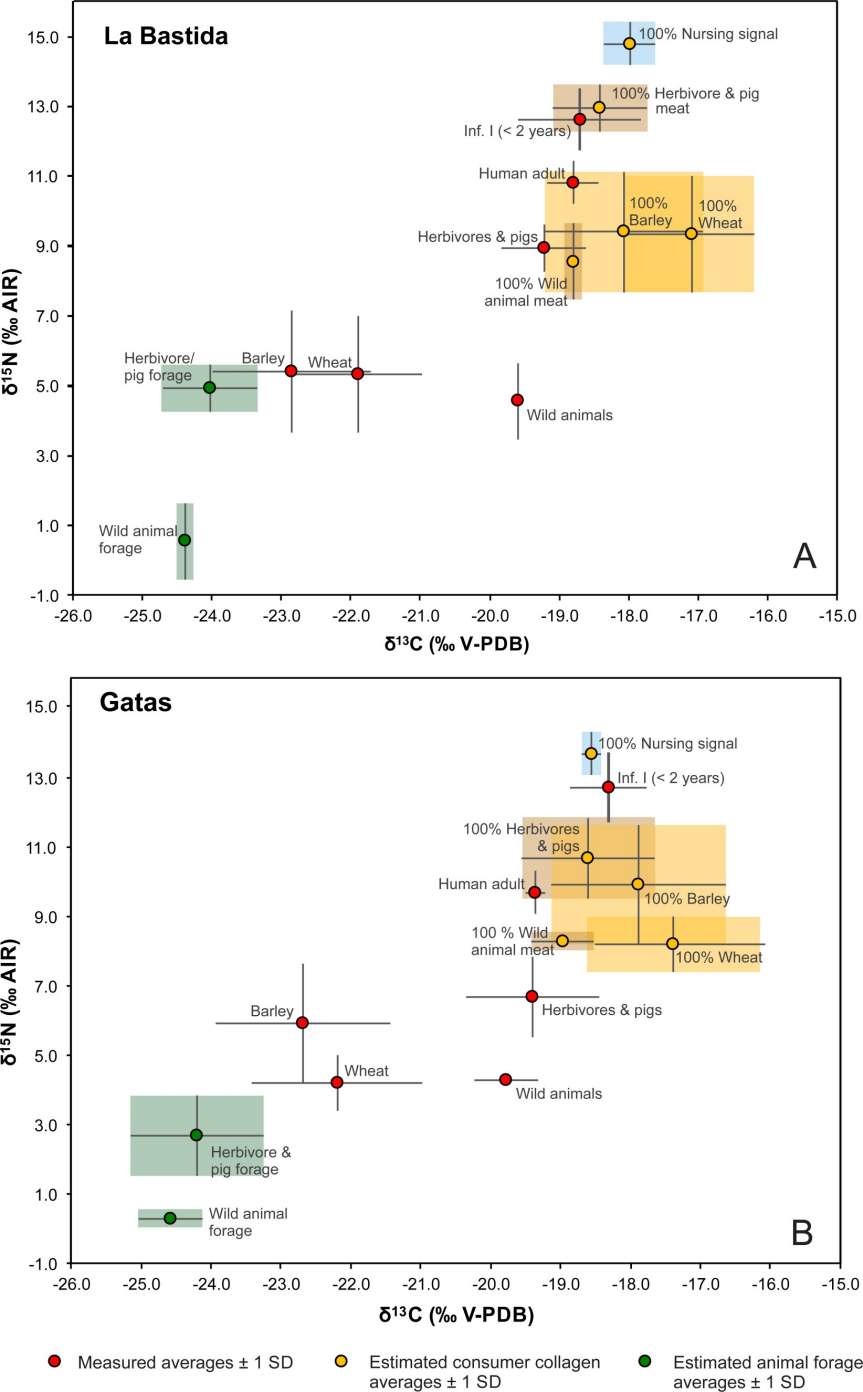
Illustration of average isotope values of the main components of foodwebs consisting of staple crops, wild and domestic animal meat and dairy products, as well as adult and infantile humans at La Bastida (A) and Gatas (B). Red symbols mark the averages of the measured values of the different data groups. Green symbols give the average isotopic composition of the forage of domestic and wild animals as estimated by subtracting typical isotopic offsets between plants and consumer collagen. Yellow symbols are estimations of the average isotopic compositions of collagen values of consumers that are one trophic level above the respective foodstuffs. All error bars and shaded fields represent one standard deviation.

Dryness and the application of animal manure are major determinants of the observed δ^15^N values of both barley and wheat grains, which were higher than those of the forage of the deer from La Bastida and Gatas as estimated by subtracting a trophic level offset of 4.0‰ from the collagen δ^15^N values of these wild animals (Fig 8; S1D Table).

This outcome indicates agricultural practices for improved soil fertility, either by the deliberate spread of animal manure, or by stubble grazing of the domestic animals on arable land. According to the δ^15^N levels of cereal grains from traditionally managed fields with similar precipitation rates as at La Bastida and Gatas, the values below 6‰ are typical for cereals that received no or little manure, while those above 9‰ indicate high manuring rates [80, Fig 4]. Compared to these estimates, the Bronze Age data suggest no/low to medium manuring rates for both barley and wheat at La Bastida and for barley from Gatas (Fig 7). In contrast, all samples of wheat kernels from Gatas indicate no/low manuring rates. The obviously variable growing conditions go along with the absence of arable land in the near vicinity of the sites. Instead, the diversity of both carbon and nitrogen isotope values indicates cultivation at different locations in the river valleys, where crops could have been grown extensively under rain-fed conditions and with little to moderate application of manure.

### Feeding of domestic and wild animals

The very similar δ^13^C and δ^15^N values of the deer collagen from La Bastida and Gatas indicate overall comparable environmental conditions at both sites (Fig 6). In contrast, the average δ^15^N values of the domestic herbivores and pigs at La Bastida are significantly higher than those at Gatas, which points to differences between the feeding strategies at both sites. Typical enrichments of the heavier isotopes along food chains indicate that the domestic animals at La Bastida were fed with plants having higher δ^15^N values than the natural food resources of the deer and with similar δ^15^N values to the charred cereal grains (Fig 8A). This suggests that the relatively high δ^15^N values of the crops contributed to the isotopic composition of the collagen of the domestic animals. A mixture of crops and their by-products as part of the animals’ forage are also in agreement with the carbon isotope values because chaff typically contributes carbon with lower δ^13^C (resp. higher Δ^13^C) values than the grains [51]. Moreover, more humid and forested habitats explain the even lower δ^13^C of the deer forage [49, 83]. Remarkably, the δ^13^C and δ^15^N values of the collagen of the omnivorous dogs are similar to those of the domestic herbivores, which indicates that crops contributed significantly to the diet of both the dogs and the domestic herbivores [84].

In sum, both the nitrogen and the carbon isotope data suggest that cereals and their by-products contributed substantially to the forage of domesticated sheep/goat, cattle and pigs at La Bastida. Depending on the season, this could have been achieved by penning the animals on harvested fields and have them eat the remnants after harvest, by stockpiling the by-products of the crop production, and even by feeding crops to the animals. Merely, the elevated δ^13^C value of one sample of a cattle bone from La Bastida (Fig 3) may indicate some contribution of C_4_ plants, which occur naturally in SE Iberia [85, 86].

At Gatas, the estimated average carbon and nitrogen isotope values of the food resources of the domesticated animals fell between those of the estimates of the wild forage of the deer and the data of the cereal grains (Fig 8B). This indicates feeding on a mixture of both crops and their by-products as well as plants from natural habitats, with a larger share of the latter than at La Bastida. Even though the sample sizes per species are low, there is indication for some variation in feeding habits of the different domesticates (Fig 5). The similarity between the isotopic composition of the deer collagen and some of the sheep and goat samples points to larger contributions of wild plants to the forage of these animals and more extensive herding strategies. The pigs had higher δ^15^N values than the sheep and goats, which may have resulted from either a larger share of crop plants or some contribution of animal-derived protein. The data agree with small ruminants grazing at the slopes of the Cabrera mountain chain where the settlement was located, while the pigs may have been deliberately fed with staple crops and their by-products. Like at La Bastida, two samples of cattle had high δ^13^C values, which probably resulted from some contribution of C_4_ plants.

### The human diet

At the first glance, the significantly different δ^15^N and δ^13^C values of the humans (Fig 6) imply that the people at La Bastida consumed remarkably more animal-derived foodstuffs than those at Gatas, an assumption that is to be tested against the isotope dataset of the crops and animal collagen.

At La Bastida, the adult individuals had on average 5.4‰ higher δ^15^N values than the charred barley grains and 1.9‰ higher δ^15^N values than the domestic animals (Fig 3; Fig 8A). Hence, the human values were somewhat higher than expected from barley consumption alone and point to some addition of animal-derived foodstuffs, such as meat and possibly dairy products. At the same time, the isotope data preclude that animal-derived foodstuffs dominated the human diet. The difference between the δ^13^C data of the domestic animals and the humans was within the typical range of the collagen offset between two adjacent trophic levels, while the offsets between the average data of both crop species and the human collagen indicates a predominance of barley over wheat. This is in agreement with the archaeobotanical record [21, 87, 88], whereas the values also suggest a certain contribution of other foodstuffs that had lower δ^13^C values than the cereals. The estimated δ^13^C values of the forage of the wild and domesticated animals, that were lower than the measured δ^13^C values of the cereal grains, indicate that such plants with possibly other edible parts than the grains were principally available in the area. The plot of average isotope compositions of the human collagen (± 1 SD) in comparison to potential consumers of 100% barley, wheat, or meat of domestic or wild animals (Fig 8A) illustrates that the isotopic composition of the human collagen could have been achieved with pure barley consumption, but is better explained by some contribution of domestic animal-derived foodstuffs. The graph also highlights that the δ^15^N values are higher than expected for a predominant consumption of wild animal meat and lower than anticipated for exclusive consumers of domestic pig or herbivore meat or dairy products.

At Gatas, the difference between the average δ^15^N values of the barley grains and the humans is 3.8‰, which resembles about one trophic level (Fig 5; Fig 8B). Therefore, a diet that was dominated by barley with some contribution of foodstuff with lower δ^13^C values than the cereal grains may have caused the observed data spectrum. However, because the domestic animals have comparatively low δ^15^N values, the consumption of meat and possibly dairy products is less well traceable at Gatas than at La Bastida. In fact, Fig 8B illustrates large overlaps of the carbon and nitrogen isotope data of pure barley consumers and consumers of a pure meat and dairy-based diet. Thus, the average isotope values of the adults at Gatas are also within the range to be expected from a predominantly animal-derived diet, although the botanical and zooarchaeological record at Gatas [87] does not supported such a situation. At the same time, the results argue against a predominance of wheat in the human diet, which should have led to lower δ^15^N and higher δ^13^C values.

Embedding the carbon and nitrogen isotope compositions of the human collagen into the context of the data of the cereals and animal collagen revealed that the significantly different δ^15^N and δ^13^C values of the humans from La Bastida and Gatas could have resulted from similar dietary compositions (Fig 6; Fig 8). Because the δ^15^N values of barely are similar at both sites, but lower for the domestic animals at Gatas, similar relative contributions of barley and meat/dairy products lead to higher δ^15^N values of the humans at La Bastida than at Gatas. At the same time, a diet that consisted completely of domestic animal-derived meat and dairy products would have led to more than 2‰ higher δ^15^N values at La Bastida than at Gatas. Moreover, the average δ^15^N value of pure meat/dairy consumers of almost 13‰ to be expected at La Bastida could not be achieved in the foodweb at Gatas.

**Fig 9:**
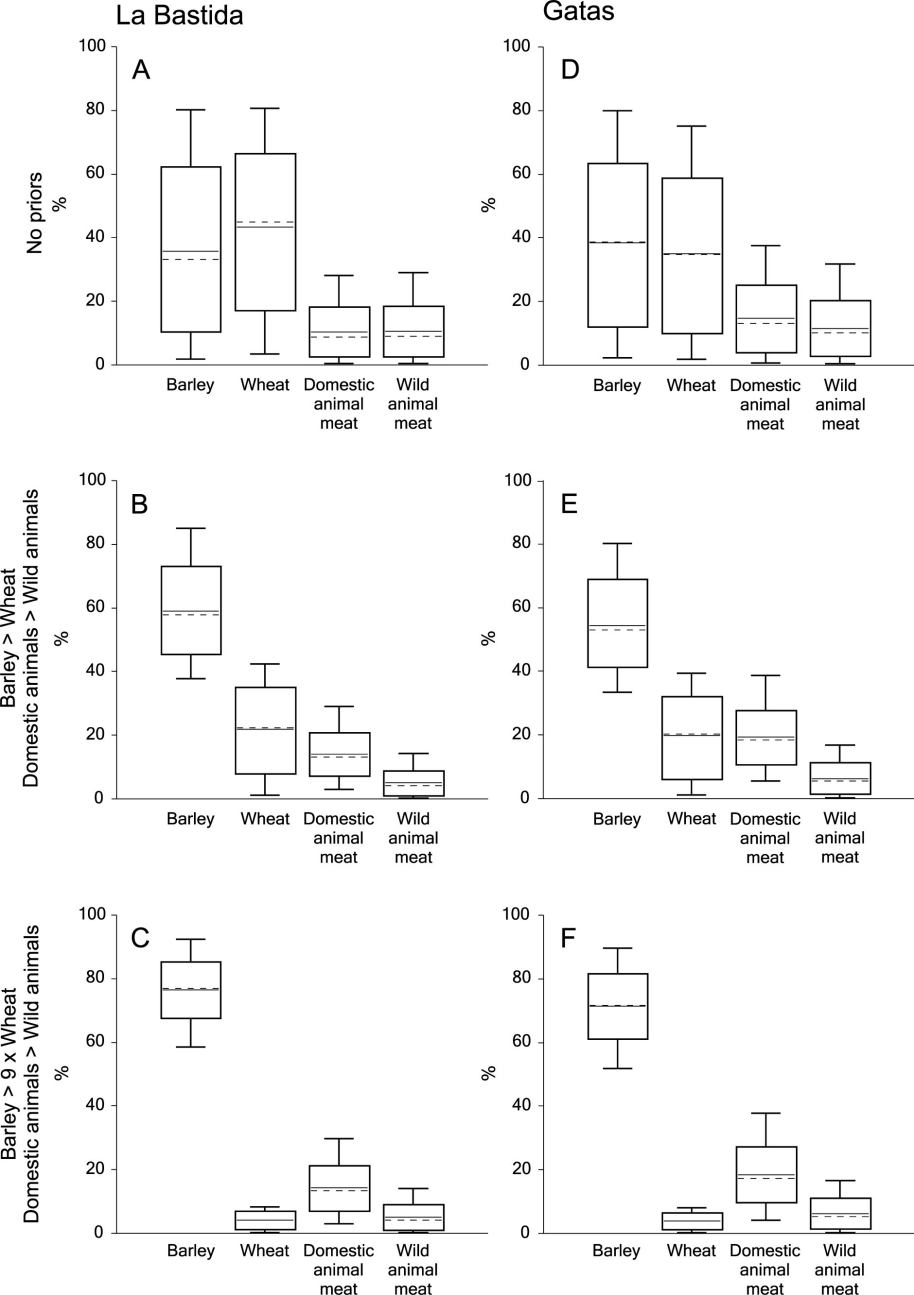
**Estimated contributions of barley, wheat, domestic and wild animal meat to the average diets of adult individuals from La Bastida (left) and Gatas (right) using the Bayesian model FRUITS.** A and D: estimations without priors, B and E: estimations under the conditions that barley outweighs wheat and domestic animal meat outweighs wild animal meat, C and F: estimations under the condition that barley makes up 90% and wheat 10% of the cereals and domestic animal meat outweighs wild animal meat.

Bayesian modelling of dietary compositions using the FRUITS program [7] also attested to similar average dietary compositions at both sites (Fig 9; S1E Table).

At La Bastida, the average contribution of cereals to the human diet adds up to 79%, whereas wild animal-derived foodstuffs made up 21%. At Gatas, these estimates were 74%, resp. 26% (Fig 9A and 9D). These approximations are, however, associated with very large uncertainties. Considering the archaeological evidence according to which barley outweighs wheat and domestic animals outweigh wild fauna [89], narrows down the possible contributions of these four considered food sources (Fig 9B and 9E). Including the archaeological evidence that barley occurs at least nine times more frequently than wheat in the middle and late Argaric settlement phases as a prior [21, 90] reduces the estimation errors further. According to these assessments, barley made up an average of 77% of the human diet at La Bastida, followed by 14% of domestic animal-derived food sources, while these numbers were 71% and 18% at Gatas (Fig 9C and 9F).

Remarkably, the FRUITS program estimates the average contributions of animal-derived foodstuffs somewhat larger at Gatas than at La Bastida, despite lower human δ^15^N values at Gatas. However, the model confirms a largely barley-based human diet at both sites and underlines that nitrogen isotope compositions of the human collagen do not *per se* reflect shares of animal-derived foodstuffs.

Because Gatas lays 3 km and La Bastida 35 km away from the coast, a possible effect of seafood must also be considered. The coastal site of Punta de Gavilanes confirms that seafaring, trading or fishing [91] must have played some role in the El Argar economy. Ancient fish samples from the western Mediterranean have δ^13^C values around -13 to -10‰ and δ^15^N values between 8 and 11.5‰ [42]. Therefore, substantial fish consumption would have resulted in both high δ^13^C (up to -11‰) and δ^15^N (up to 14‰) values of the human collagen. Especially the carbon isotope data argue against a significant contribution of marine fish, despite the proximity to the Mediterranean Sea.

### Early childhood diets

Subadult individuals made up a significant portion of El Argar burial communities [92–94], of which La Bastida and Gatas are representative examples with 39% resp. 49% of the excavated individuals belonging to the age groups infans I and infans II. Similar observations were usually interpreted as evidence for relatively high neonatal death rates and infant mortality [92, p. 269, 93, 94, p. 66]. However, Argaric children and adults received similar mortuary treatments from *ca*. 1900 cal. BCE onwards, which stands in contrast to many other prehistoric contexts, in which skeletal remains of subadult individuals are underrepresented. Therefore, the significant proportion of children among the burials investigated here does not necessarily point to extraordinary high mortality rates, which may have resulted from childbirth complications, low birth weight, infectious disease, and malnutrition (dietary stress).

**Fig 10:**
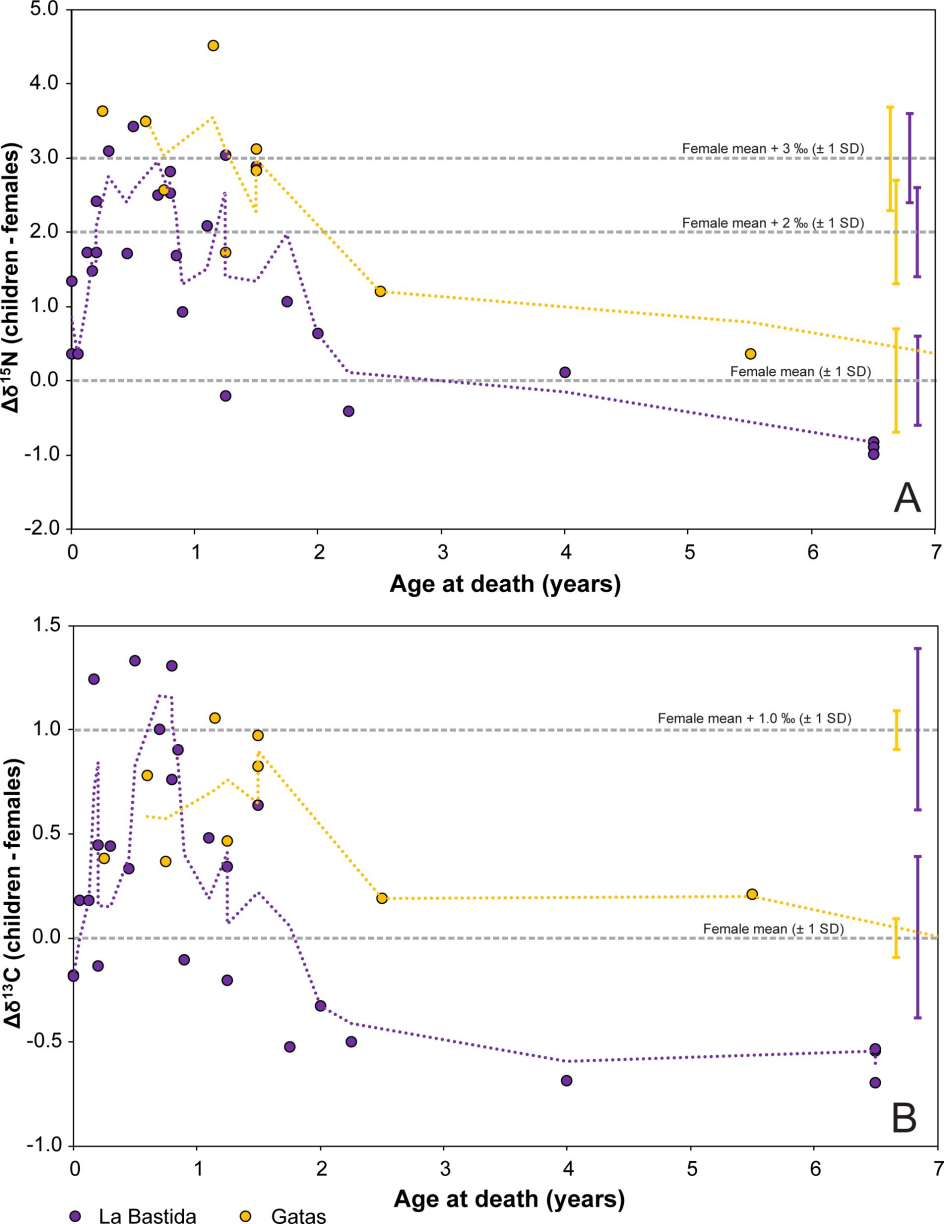
Nitrogen (A) and carbon (B) isotopic offsets between the children from birth to seven years of age and average values of the females from La Bastida (purple) and Gatas (yellow). The horizontal lines and error bars mark the female averages ± one standard deviation and the value 2‰, resp. 3‰ above it, a range that is widely considered typical for breastfed children [65, 67]. The dotted lines are moving averages of the isotope values of the children.

Despite the caveats of cross-sectional studies [67], the considerable datasets of bone collagen of infans I and infans II individuals have some implications on early childhood diets. At La Bastida, δ^15^N values of the 23 infans I individuals increased between birth and 0.5 years from values slightly above the female average to maximally 3.4‰ above it (BA 26) (Fig 10). Most samples of individuals who died until around 1.5 years yielded elevated δ^15^N values of up to around 3‰ above the female average, whereas the values decreased between 1.5 and 2 years towards the range of the females. There was a similar trend of increasing average δ^13^C values in the first months and overall elevated levels in the first 1.5 years, despite more variation relative to the total range of the data. Similar to other studies, the δ^13^C values dropped earlier than the δ^15^N values [95].

The infants from Gatas also exhibited a similar trend (Fig 10). All seven individuals between 0 and 1.5 years of age had δ^15^N values considerably above the female average, with a maximal difference of 4.5‰ (GA 21). The same samples exhibited δ^13^C values above the double standard deviation of the females and four of them were in the range of about 1‰ above the female average (± 2 SD). At Gatas, the alteration of the isotope signals over time was less well documented due to a lack of neonates and children who died around two years of age. A skull sample of a child of two to three years of age still exhibited an elevated δ^15^N value (GA 38). As stated above, the interpretation of these data regarding the duration of breastfeeding in El Argar communities lacks precision. The incorporation of the dietary isotope signals is delayed due to bone turnover, possibly interrupted due to stunting, and the diet of the prematurely deceased individuals may have differed from those who survived into adulthood. At least at La Bastida, children of two years and older did not exhibit any increased δ^15^N and δ^13^C values anymore, which indicates that breastmilk consumption has ceased to be the dominant food source of children before that age. At Gatas, the slightly elevated δ^15^N value of the two to three year old individual GA 38 does not necessarily imply prolonged breastfeeding, because skull bones have slower turnover rates and may retain the nursing signal longer than ribs that were sampled for most other individuals [62, 96, 97].

Remarkably, the differences between the average δ^15^N and δ^13^C values of the children up to two years old and the females were considerably larger and less variable at Gatas (Δδ^15^N_children < 2 years–females_: 3.1 ± 0.9‰; Δδ^13^C_children < 2 years–females_: 0.7 ± 0.3‰) than at La Bastida (Δδ^15^N_children < 2 years–females_: 1.8 ± 1.0‰; Δδ^13^C_children < 2 years–females_: 0.4 ± 0.6‰). Consequently, despite lower mean δ^15^N values of the females at Gatas than at La Bastida (9.5 ± 0.7‰ vs. 10.8 ± 0.6‰), the δ^15^N values of the children below two years of age were very similar (12.6 ± 0.9‰ at Gatas; 12.7 ± 1.0‰ at La Bastida) (Fig 6; Fig 8). Δδ^15^N_children < 2 years–females_ around 3‰ or above may not only document breastfeeding, but also result from metabolic stress due to which the human body metabolizes its own proteins [65, 68]. Samples of child bones with high turnover rates of 300% during the first months of life and 100% at one year of age [66] may have been able to record the associated changes in the isotope composition of the collagen shortly prior to the death of the individuals. The higher Δδ^15^N_children < 2 years–females_ values at Gatas than at La Bastida indicate that children at Gatas possibly suffered from more intense or prolonged metabolic stress.

The more variable and often smaller offsets between the isotope data of the young children and the females at La Bastida imply both, more variability of the dietary habits of the mothers and probably an earlier introduction of supplementary foods that likely consisted of C_3_ cereals and/or milk of ruminants. The latter is problematic in case of cow’s milk, which has higher protein and mineral contents, but lower shares of lactose than human breast milk [98]. Some of the δ^15^N values of the bone collagen of the children below 1.5 year of age are less than 2‰ above the average value of the females. They probably do not represent pure nursing signals, but either a mixture between the in-utero-signal of the mother and breast milk, or a mixture of breast milk and supplementary foodstuffs, which increasingly outweighed the breast milk signal from 1.5 years on.

Overall, the data document nursing signals–or metabolic stress–in children up to 1.5 years old at both sites and imply the cessation of breastfeeding around two years at least at La Bastida. In historical periods, mortality rates often depended on food supply and were particularly high if breastfeeding periods were very short (< 6–12 month) [99, 100]. On the other hand, breastmilk alone does not meet the nutritional and energetic requirements of children from around 6 months on [65], and an exclusive continuation of breastfeeding until the age of 2 years is not only unlikely, but also unhealthy [67]. Being aware of the uncertainties regarding the information of cross-sectional data, there is no indication of any prolonged average breastfeeding periods at La Bastida and Gatas, whereas the early transition to solid foodstuffs and cessation of breastfeeding may remain invisible due the low temporal resolution of the data.

### Differentiation among the adult population

The very similar stable isotope compositions of males and females at both sites imply that sex was not the primary driving factor of the internal differentiation of the datasets and that both sexes had similar access to the available resources (Fig 3; Fig 4A and 4D; Fig 5). Especially at La Bastida, where cereal and animal-based diets were more isotopically distinct and the human dataset appears more variable, sex-specific dietary habits should have been detectable. The similar isotope compositions of samples of both sexes agree with the archaeological record of typical Argaric intra-mural funerary practises and also do not preclude varying proportions of isotopically similar foodstuffs.

Rather standardized grave goods, or the lack of them, reflect social differences in the funerary ritual [12]. The isotope data from La Bastida suggest that social categories as manifested in the archaeological record went to some extent along with dietary differentiation in lifetime. All three individuals assigned to the highest social category (females BA 60 and BA 40/1, and male BA 40/2) and one representative of the next lower category 3 (male BA 12/1) have both elevated δ^13^C and δ^15^N values (Fig 4B).

Especially the higher δ^15^N values point to larger shares of animal-derived proteins, and therefore the consumption of overall higher-quality and presumably more valued food. The female BA 83 with the highest δ^13^C value and an average δ^15^N ratio belonged to the lowest social category 5. The combination of isotope values found in her bone sample may indicate that wheat, with on average higher δ^13^C values, outweighed barley as the otherwise predominant crop. Moreover, some contribution of C_4_ plants to her diet seems plausible. The individuals with the lowest δ^15^N values at La Bastida (BA 18/2 and BA 48) belonged to the social categories 3 and 3–4. Possible explanations of this pattern include a diet that was especially low in domestic animal-derived protein, contribution of wild animal protein or an origin in another community with overall lower isotope values, such as those found at Gatas. All remaining individuals, and therefore most of the burial population, form a central data group that comprises burials of the lower social categories 3–4, 4, and 5 without any further differentiation. In this case, differences in the funerary ritual do not correlate with dietary habits, suggesting that access to food resources was rather uniform for the vast majority of the population.

In the smaller dataset of adult individuals from Gatas, indications of social differentiation were not traceable. Only, the male GA35/2 of a rich but disturbed burial of the final moments of El Argar exhibited the highest δ^15^N value. The halberd-bearing male GA 41, who represented the highest social category during an early phase, was not distinguished by his isotopic values. There was also no indication for a diet-related separation among the lower social classes. However, at Gatas, barley grains and collagen of domestic animals exhibited stable isotope compositions that do not distinguish well among cereals and animal-derived foodstuffs, leading to ambiguity in data interpretation at this site.

Some tendencies also arise regarding the chronological differentiation (Fig 4C and 4F). At La Bastida, all individuals that formed the cluster of higher δ^15^N and δ^13^C values belonged to the older chronological phase (c. 2000–1750 cal. BCE), while both individuals with the lowest isotope values were assigned to the younger chronological group (c. 1750–1550 cal. BCE). The significantly lower δ^15^N values in the younger phase may indicate a dietary shift over time that comprised either decreasing shares of animal-derived protein or a decrease of the δ^15^N values of the cereals and meat itself. The latter would point to an extensification of agricultural strategies and losing bounds of cultivation and animal husbandry, being connected with less manuring and less contribution of staple crops and their by-products to the forage of the domestic animals. It is too early to discern whether these data are a first hint on an isotopic support of the hypothesis that El Argar culminated in an overexploitation of the agricultural resources that lead to a decrease in food quality and quantity, elevated child mortality and finally the end of the socio-political organisation [18]. The dataset from Gatas was too small and inconclusive regarding changing dietary habits over time.

### Comparison to other sites

**Fig 11:**
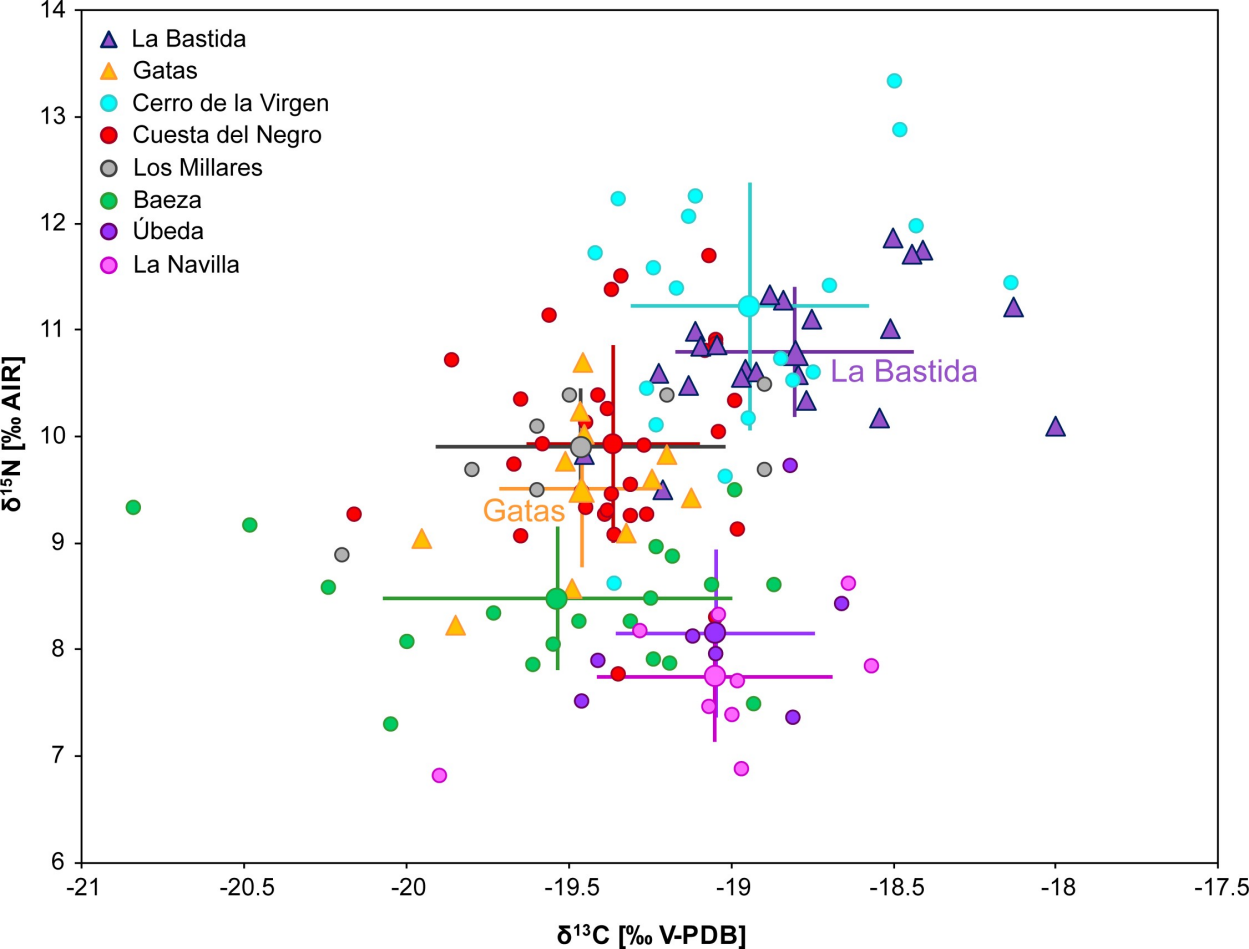
Carbon and nitrogen isotope compositions of individuals above 14 years of age from La Bastida, Gatas and published datasets from Bronze Age sites in southeastern Spain. Large symbols and error bars mark the averages and one standard deviation of the respective datasets. (Data: [101–103]).

Putting the results of the stable isotope analyses from La Bastida and Gatas into the context of previous analyses on human remains from prehistoric southeastern Iberia revealed a considerable variability, which seems to correspond to chronological, ecological, and economic differences (Fig 11).

In particular, the δ^13^C and δ^15^N values of the individuals from La Bastida (excluding age categories infans I and infans II) are among the highest of all data from Argaric contexts and very similar to those from the settlement of Cerro de la Virgen [101], which is located on a terrace overlooking a fertile valley, in inland Southeast Iberia. The on average lower δ^13^C and δ^15^N values at Gatas overlapped widely with those from Copper Age Los Millares [102] and Argaric Cuesta del Negro [103]. While the first two are situated close to the Mediterranean shoreline, Cuesta del Negro is a hill-top settlement of the inner Southeast, not far from Cerro de la Virgen. Remarkably, the somehow different Argaric sites of Baeza and Úbeda [103] form a group with the lowest δ^15^N values, together with the Neolithic passage grave of La Navilla. Baeza and Úbeda represent large settlements dominating a rolling landscape of the upper Guadalquivir river valley, while La Navilla belongs to a megalithic necropolis in southwest Granada (Fig 1). If we only focus on El Argar settlements and assume that δ^15^N primarily reflects husbandry practices and manuring rates, as identified in Gatas and La Bastida, the inhabitants of the large upper Guadalquivir site of Baeza, enjoying today *ca*. 500 mm of annual rainfall, did not improve soil productivity artificially and seem to have practiced comparatively extensive animal husbandry. In contrast, more intensive manuring, would have prevailed in the relatively small (*ca*. 0.7 ha) settlement of Cerro de la Virgen, with a current annual rainfall of approximately 370 mm, as well as in the *ca*. 5 ha large town of La Bastida, with about 270 mm annual rainfall. Gatas and Cuesta del Negro, with *ca*. 250 and 480 mm annual rainfall respectively, would have practiced manuring and husbandry with intermediate intensity. However, reading the different δ^15^N values as a reflexion of the shares of animal-derived proteins to the human diet has very different implications. Therefore, overcoming the interpretative ambiguity requires extending the isotope analyses at the other sites to include more members of the trophic chain, particularly bones of different animal species and cereal grains.

## Conclusions

La Bastida and Gatas are two representative and well investigated hilltop settlements of the El Argar society, which were inhabited between 2200 and 1550 BCE. While La Bastida was a fortified urban centre with possibly *ca*. 1000 people, who controlled the resources of a larger region, Gatas represents a much smaller site, with probably not more than 300 inhabitants. The carbon and nitrogen isotope data confirm that the economy of El Argar was firmly based on cereal agriculture, supplemented with a certain amount of animal resources, as suggested by botanical and faunal evidence, and most of all by the accumulations of grinding slabs, often in specific workshops. Evaluated in the context of data from cereal grains and animal collagen, the isotope data of the human bones suggest similar subsistence strategies of the populations at both settlements. During the latest phase of the El Argar culture, either the contribution of meat and dairy products or manuring seem to have decreased, implying a decrease of the agricultural yields. However, more samples are needed to confirm the archaeologically hypothesized subsistence crises, which likely caused the abrupt end of the highly centralised political and social organisation.

The isotopic data also suggest strong interrelations between crop cultivation and animal husbandry. Stockpiling, grazing on stubble fields after the harvest and possibly even the direct feeding of grains most likely contributed significantly to the domestic animals’ forage. Reversely, dung provided valuable manure enhancing the fertility of the agricultural plots. This interrelation was stronger at La Bastida than at Gatas and confirms the economic force of this urban settlement, which likely depended on the intensive agriculture and husbandry practised in the fertile Guadalentín valley, and not on resources of the mountain environment where the settlement was located. Smaller population sizes might explain why this more intensive subsistence strategy was not practiced in Gatas and other Argaric sites. The overall lower and more variable δ^15^N values at Gatas and the evidence for C_4_ plants in some of the cattle’s forage also point to diversity among the grazed habitats and pastures in this settlement. Whether some of the variability resulted from being temporarily away from the sites, like it is practised in transhumance or transtermitance, requires further investigations such as strontium isotope analysis on animal tooth enamel.

Cross-sectional evaluation of the stable isotope data indicates that children at both sites were breastfed until an age of 1.5 to 2 years. Among those who died prematurely, infants from Gatas may have suffered from more intense or prolonged metabolic stress than those from La Bastida. Moreover, the isotope data reflect social differences inferred from the funerary record and the economic organization of the settlements to some extent. At La Bastida, individuals buried with the most distinguished grave goods, which point to being members of the dominant class, likely enjoyed a diet with larger shares of animal-derived proteins. However, according to the isotopic compositions of the different sample materials, especially at Gatas, foodstuffs of animal and plant origin are not always isotopically well distinguishable. Moreover, the selection of certain portions of meat and food preparation determine the quality of food significantly, but are not reflected in the isotope data. Therefore, the differentiation of the dietary habits of the Argaric population may have gone far beyond what is reflected in the stable isotope composition of the human bone collagen, and further analyses are required to add additional nuances to our understanding of the everyday implications of economic exploitation and social differentiation in El Argar.

Importantly, this study has shown the relevance of considering complete trophic chains to interpret the stable isotope record of human remains adequately. Human bone data alone would have suggested that dietary habits at both investigated sites differed significantly, and the inhabitants of La Bastida enjoyed larger shares of meat and dairy products indicating economic and/or political superiority over those at Gatas. However, extending the analysis to grains and animal bones showed that the differences were caused by the economic practices and, more specifically, by the closer management of agriculture and husbandry in the urban centre. The present study therefore also emphasizes the methodological complexity of interpreting carbon and nitrogen isotope data and the importance of viewing human isotope data in the context of comparative data and the archaeological setting.

## Supporting information

S1 Table Data. A) Carbon and nitrogen contents and isotopic compositions of human bone collagen from the sites of La Bastida and Gatas and context information on the respective burials. B) Carbon and nitrogen contents and isotopic compositions of animal bone collagen from the sites of La Bastida and Gatas. C) Carbon and nitrogen contents and isotopic compositions of charred cereal grains from La Bastida and Gatas. D) Average δ^13^C and δ^15^N values of subgroups from La Bastida and Gatas. E) Bayesian modelling of dietary compositions using the FRUITS program. Data entry and results.
(XLSX)
